# Role of social capital in adolescents’ online gaming: A longitudinal study focused on the moderating effect of social capital between gaming time and psychosocial factors

**DOI:** 10.3389/fpsyg.2022.931134

**Published:** 2022-08-09

**Authors:** Gyoung Mo Kim, Eui Jun Jeong, Ji Young Lee, Ji Hye Yoo

**Affiliations:** ^1^Department of Digital Culture and Contents, Konkuk University, Seoul, South Korea; ^2^Ministry of Culture, Sports and Tourism, Sejong, South Korea

**Keywords:** online gaming, social capital, psychosocial effects, adolescent gamers, longitudinal study

## Abstract

Adolescents often create social relationships with their gaming peers who take on the role of offline friends and peer groups. Through collaboration and competition in the games, the social relationships of adolescents are becoming broader and thicker. Although this is a common phenomenon in online games, few studies have focused on the formation and roles of social capital among adolescent gamers. In particular, longitudinal research that examines the role of social capital in terms of influencing gaming time on adolescent gamers’ psychosocial factors has been minimal. This study was designed to fill this gap to see the long-term effect of social capital among adolescent gamers. Specifically, by using the three-year longitudinal data involving 403 adolescents, we analyzed the effect of gaming time on psychological factors (i.e., loneliness, depression, self-esteem, and life satisfaction) with the moderating role of social capital. Results showed that social capital played a crucial moderating role. In the higher social capital group, gaming time enhanced the degree of self-esteem and life satisfaction. However, a vicious circle was found in the lower social capital group: Gaming time increased the degree of depression but decreased self-esteem, which in turn led to increase in gaming time. These results indicate that games work as an important tool for social capital cultivation among adolescent gamers, which imply successful cultivation of social capital is a key to positive gaming effects. Theoretical and practical implications are discussed.

## Introduction

Social network creation and formation of social capital (i.e., personal benefits through relationship) have been discussed as among the most important aspects of online games (Kolo and Baur, 2004; Taylor, 2006; Ju and Lee., 2018; Perry et al., 2018). As games are increasingly played online, communication activities with other players account for a large part of players’ activities in a game (Suznjevic et al., 2009). These communication behaviors include having a conversation to cooperate with other game players, helping other game players, and engaging in activities to lead the group and accomplish the goal. These behaviors are aligned with social interactions, which are a motivation in online games (Yee, 2006). As a means of self-expression and social interactions with others, games can play an important role in predicting and nurturing a user’s psychosocial characteristics and social relationship (Ducheneaut and Moore, 2004; Brian and Hastings, 2005; Cole and Griffiths, 2007; Zhong, 2009).

Considering that adolescence is a time when young people expand their relationships with others, the role of games in social interaction could peak during this time. In this period, adolescents experience physical, cognitive, and emotional changes internally, and various social relationship changes externally as the scope of their social activities expands (Engels et al., 2002). Acquiring social capital through appropriate interactions in this period can be the element that positively affects adaptive behavior during adolescence (Jung, 2022).

Even though playing online games is fundamentally a social activity among adolescents, the effects of social capital among adolescent gamers were rarely proven empirically. It was because many scholars had not agreed to consider that online games were a place for activities such as sharing emotional support with peers, discussing issues, and expanding the network (Ju and Lee, 2018). The social capital formed through online games has value as a study reflecting the peer culture during adolescence.

Many studies have examined the relationship between the use of games and individuals’ psychosocial well-being, producing various findings (Griffiths and Hunt, 1995; Ballard and Wiest, 1996; Anderson and Bushman, 2001; Durkin and Barber, 2002; Lager and Bremberg, 2005; Young, 2007; Abreu et al., 2011; Grizzard et al., 2014). There are only a few longitudinal studies on the use of games and early adolescents’ psychosocial factors, especially ones related to social capital. Nevertheless, the longitudinal study is a further attempt to view the social capital accumulated through playing games as a critical factor affecting adolescents’ psychosocial characteristics (depression, loneliness, life satisfaction, and self-esteem).

This study examined the impact of games on the cultivation of adolescents’ social capital and psychosocial variables and explores how adolescents utilize their social capital to manage psychological status through longitudinal empirical data. Consequently, this supplements the shortage of previous research and social capital’s impact on adolescents’ psychosocial factors and change in gaming play behavior.

## Literature review and analysis framework

### Social capital and online gaming

*Social Capital* is defined as an attribute that makes specific actions available within a social structure (Coleman, 1998), including personal relationships and benefits that go with them (Williams, 2006). Social capital has been reported to improve the community’s efficiency by promoting collaborative behavior among individuals in society. Thus, various fields have applied it to communication and networking. Likewise, since social capital is conceptualized as a network of shared dependencies and obligations within a society, it has been applied to the field of societal problems (Weiss, 2011).

Individual social capital includes two aspects of social capital-social bonding and social bridging (Trepte et al., 2012). Social bonding implies accumulated social capital in a network and expected benefits from strong ties such as emotional and financial support. Social bridging refers to social capital with benefits or relationships from weak links, and it encompasses connectedness with members in a different group (Pénard and Poussing, 2010). A group with solid degrees of social bonding reveals personalities that show internally condensed socialization rather than external communication, while a group with high levels of social bridging creates cooperative communities of diversity in gender, age, and race.

The core figurative requirements of social capital consist of trust based on reciprocity and social relationship in a network (Putnam, 2000). Trust based on reciprocity is the belief that a community member (or organization) responds in good faith. The social network in a society serves as a pathway for amicable relationships to provide and receive solid support (Castillo, 2019).

In this research, the concept of social capital is referred to as the achieved experience of acceptance and belonging that enable adolescents to interact. Since the core need lies in the innate human tendency to gain acceptance and avoid rejection (Hladik and Hrbackova, 2021), peer relationship plays a distinct developmental role during adolescence (Sullivan, 1953; Rudolph, 2020).

Compared to offline groups, online communities can build social relationships and build reciprocal trust because people usually participate voluntarily in online communities (Perry et al., 2018). Owing to these characteristics, many studies have considered the influence of social capital on the Internet or social media users (Williams, 2006; Yoo and Jeong, 2017). However, research on social capital foundation and influence through online game use remains insufficient. Research on whether online game players form social capital through gameplay or positive or negative social capital effects through online games is still minimal.

Online games provide rich virtual environments that enhance a wide range of social experiences (Ducheneaut and Moore, 2004; Brian and Hastings, 2005; Cole and Griffiths, 2007; Zhong, 2009; Kaye et al., 2017). Through player-to-player cooperation and competition, players follow the norms within the group. These conduct activities are aligned with the group’s interest (Gong et al., 2019) and the players interact socially. Social interaction is “the process of reflecting on relationship with people around us” (Giddens, 2001), and online games function as a virtual reality space where social capital is formed based on active social interaction (Lo et al., 2005; Yee, 2006). Online games provide players with a teamwork experience to win in-game battles and communicate to solve problems and allow players to share information about gameplay and recent game achievements (Arbeau et al., 2020).

Adolescence is when peer relationships become the center of social relationships, so it is common for adolescents to spend less time with parents and more time with peers (Kim et al., 2019). For adolescents, online game play is one of their playing cultures, and it becomes the most crucial space where they make friends and experience peer attachment. Social support from peers has a tremendous effect on adolescents’ self-esteem development and life satisfaction. It could also be inferred that social capital influences adolescents’ psychosocial well-being.

### Online gaming and psychosocial well-being

Based on the claim that media use affects players’ perceptions or behaviors and forms a strengthened usage pattern (Slater, 2007), online games can create social capital based on the degree of involvement in games and a players’ usage habit change. Online game social capital formation is highly related to players’ habits and motivations (Reer and Kramer, 2019). The results of an empirical study that players’ social capital and psychological factors could be affected (Williams, 2006) supports the claim mentioned above.

Social support through the formation of social capital reduced the player’s sense of loneliness (Snodgrass et al., 2019), while the achievement and failure in terms of the player’s psychosocial needs could be an essential factor in game immersion (Weinstein et al., 2017). In this regard, it is meaningful to analyze the influence of social capital formation experience on online game use and the psychosocial factors of players.

Psychosocial factors demonstrated by the players were relatively common when the themes are related mainly to excessive game use, game addiction, or problematic game use. Most previous studies showed that the amount of gaming time influenced players’ depression and loneliness (McKenna and Bargh, 2000; Nie et al., 2002; Cheung and Wong, 2011; Lemmens et al., 2011; Guo et al., 2012; Wei et al., 2012; Ko et al., 2014). Controversies about these results still exist, but depression and loneliness are representative of the psychosocial factors (Carras et al., 2017). Also, as a recent study revealed that levels of need satisfaction in games cause online game players’ real-life satisfaction (Allen and Anderson, 2018; Fazeli et al., 2020), with life satisfaction chosen as one of the psychosocial well-being factors. The unfulfilled needs caused depression and loneliness, and their change would affect life satisfaction.

Most studies involving adolescents considered self-esteem as one of the psychosocial factors in general, and this is because self-esteem is important during adolescence. Self-esteem established in this period lasts for the rest of their lives and if they consider themselves as worthy, then self-esteem rises (Banstola et al., 2020). It is also worth noting that self-esteem is vulnerable to social exclusion or rejection of insufficient social capital (Arslan, 2019). Therefore, this research included self-esteem as one of the most essential psychosocial well-being factors among adolescents.

#### Depression and loneliness

Depression refers to a state where one is constantly sad and loses interest in activities (Association, 1997). It has been reported to strongly affect interpersonal relationships, and people who suffer from it can try to fight it by developing their social life and forming interpersonal relationships. Numerous studies report that too much online gameplay increases adolescents’ depression (McKenna and Bargh, 2000; Nie et al., 2002; Ko et al., 2014). Some studies have shown that excessive game use can increase depression and anxiety levels (Cheung and Wong, 2011; Guo et al., 2012; Wei et al., 2012). Several types of research have accentuated the positive relationship between depression and online game dependence (Peng and Liu, 2010; Griffiths et al., 2017; Chang and Lin, 2019). Fazeli et al. (2020) also demonstrated that depression was a potent mediator between excessive game playing and quality of life. Based on these previous studies, it can be stated that depression is an essential factor that relates to online gameplay directly or indirectly.

Loneliness is typically defined as the cognitive awareness of a deficiency in one’s social and personal relationships and the ensuring affective reactions of sadness, emptiness, or longing (Asher and Paquette, 2003). Various studies also show an association between online games and loneliness (Kim et al., 2009; Lemmens et al., 2011; Jeong et al., 2017). Hussain and Griffiths (2009) confirmed that adolescents who played games repeatedly felt more isolated and lonelier. Seay and Kraut (2007) showed that playing games directly increased adolescents’ loneliness. Lemmens et al. (2011) also suggested that lonely individuals are more likely to engage in games excessively. Over time, problematic use of this medium contributes to increased levels of loneliness.

In contrast, Chappell et al. (2006) suggested that playing an online game is an effortless, speedy, and inexpensive way to socialize and avoid feelings of loneliness. When gamers were actively involved in their community activities, loneliness was not related to gaming time (Carras et al., 2017). Although some results of previous studies are controversial, loneliness certainly has been viewed as one of the most valuable factors in the relationship between online gaming and players’ psychological needs.

#### Life satisfaction and self-esteem

Life satisfaction is a general evaluation of one’s quality of life according to a personally chosen set of criteria. It refers to the level of satisfaction with one’s current state and a cognitive appraisal of how satisfying one’s present life is, based on one’s previous life experiences (Shin and Johnson, 1978). While studies focus on whether online games have a positive (Kim et al., 2005) or negative (Rasmussen, 2000; Shapira et al., 2000; Wang et al., 2008) effect on life satisfaction, the conclusions are inconsistent. Recent research focused on levels of need satisfaction in online games, which affected the levels of real-life satisfaction (Allen and Anderson, 2018). In Putnam’s (2000) opinion, social connectedness is an influential factor determining happiness, and the relevance between online games with much social interaction for the gratification of needs and players’ life satisfaction can be inferred.

Self-esteem is the subjective evaluation a person makes and maintains about oneself and the extent of belief in one’s capability, worth, and significance conveyed through their attitudes and verbal behavior (Wilson et al., 2010). Furthermore, self-esteem is considered a critical psychosocial factor during adolescence. Thus, the number of studies on the relationship between online games and adolescents’ self-esteem is growing. Self-esteem directly affects online games or online addiction, and research indicates that self-esteem harms game addiction and adolescents addicted to games have low self-esteem (Armstrong et al., 2000; Ko et al., 2005; Niemz et al., 2005). In contrast, some studies emphasize that when adolescents earn a high level of satisfaction through games, they have a higher level of self-esteem than those who do not (Lee and Jeong, 2015).

Self-esteem is also affected by experiencing social support and exclusion. Social support can help increase self-esteem, but social exclusion can hurt self-esteem and prevent growth (Lin et al., 2018). People encourage themselves to decrease the chance of rejection and exclusion by gaining reciprocal trust and building social networks.

Adolescents also want to build and maintain self-esteem through psychological and social support from peers (Gorrese and Ruggieri, 2013; Shin et al., 2017). Positive self-esteem formed during adolescence increases life satisfaction (Baumeister et al., 2003) while negative self-esteem hurts psychological and physical adaptation (Sowislo and Orth, 2013), so peer group and social interaction within it is a vital variable during adolescence (Kwon and Kim, 2019).

### Analysis framework and research questions

Slater (2007) applied social cognitive theory (Bandura, 2001) to propose a “reinforcing spirals approach” composed of three stages to understand the complex interaction between the player’s psychosocial factors and media effects. The three stages are as follows: (1) use of the media affects players’ cognition or behavior; (2) players’ affected cognition or behavior affects the use of the media; and (3) repeating this process is reinforced reciprocally over time. Researchers who demonstrated a reinforcing spirals pattern for mutual reciprocity of media use and psychosocial well-being substantiated this process (Slater, 2007; Slater and Hayes, 2010; Lemmens et al., 2011).

This study proposes a reciprocal effect between online game use and the player’s psychosocial factors based on the reinforcing spiral model. Therefore, this study used SCT to test the reciprocal relations between online games and psychosocial factors depending on online social capital. Defined by the level of online social capital, the following assumptions were made: (1) online game use will influence personal psychosocial factors (positively or negatively); (2) psychosocial factors will in turn influence online game use; and (3) continuous repetition of the processes will reinforce the reciprocal relations among the variables.

This repeating process will demonstrate the mechanism of mutual influence among social capital, psychosocial well-being factors, and online gaming. Under the assumption, accumulated social capital in online games functions as peer groups considering the characteristics of the developmental cycle of adolescents. Thus, the level of amicable relationships with peers influences the psychosocial factors of game players. Ultimately, these effects will lead to change in game use or specific patterns of playing.

Social capital catalyzes online gameplay and adolescents’ psychosocial characteristics. Furthermore, the relationship between the use of online games and psychosocial factors varies depending on the level of social capital (increased or decreased). In this regard, we proposed the following research questions.

RQ1. What is the longitudinal effect of online games on adolescents’ psychosocial factors?

RQ2. Is the relationship between T1 variables (online gaming time and psychosocial variables) and others (T2 and T3 variables) moderated by cultivated social capital? In other words, is there a moderating effect depending on the level of social capital (increased or decreased)?

## Materials and methods

### Sampling and data collection

To analyze the causation and cumulative effects of social capital, psychosocial factors, and online games, this study used a panel survey with a professional survey research company in South Korea^1^: Two years of panel data were used to carry out the longitudinal study. A panel survey was conducted involving adolescents (aged 14–16 years) with whom the survey was conducted 3 times over the course of 3 years (T1, T2, T3: one-year interval) to acquire longitudinal data. Primary respondents included 710 individuals, among whom 592 participated in the second round, and 461 in the third round. A total of 403 individuals, excluding missing values, were selected for the final group.

In the first round of survey results, 362 (51%) of the respondents were male, and 348 (49%) were female. In the second round of survey results, 293 (49.5%) respondents were male, and 299 (50.5%) were female. In the third round of survey results, of the 403 selected participants, 202 (50.1%) were male and 201 (49.9%) were female. The 403 participants were subsequently divided into two groups based on social capital levels and were examined. Differences between participant social capital scores between T1 and T3 were divided into two groups, one group with social capital increase and one group with social capital decline, using a median split in order to investigate whether participants had experienced a change (or maintenance) in social capital during the two years (Trepte et al., 2012). Accordingly, participants were placed in the lower social capital group (*n* = 169) if the difference in their social capital scores were below 0.0, while participants were placed in the higher social capital group (*n* = 234) if their social capital scores exhibited no difference or were above 0.0. The median split of this study was 0.00, with 169 participants included in the lower social capital group and 234 included in the higher social capital group.

### Analytical strategy

A structural equation modeling (SEM) and repeated measures analysis, which uses the GLM (General Linear Model), were used to verify the research questions. Because this study is composed of dichotomous factors (social capital level: higher and lower), the repeated measures GLM can test the meaningful influence of social capital levels and change over time. It is also an appropriate method to measure whether time functions as a factor regulating social capital. This study utilized SEM to estimate and analyze the cause and effect between subject factors, or the entire panel. The GLM was supplemented with the SEM analysis, which increased the estimation efficiency that considers time-sequential characteristics. The SEM also presented extremely useful results for analyzing the correlations between factors from the perspective of the entire model.

### Measures

*Social capital* was measured using Internet Social Capital Scales (ISCS; D. Williams, 2006), with each question measured on a scale of 5 points (1 = not at all; 5 = very much so). Using a questionnaire of 20 items, 17 items were selected through a reliability analysis (e.g., “People who are interactive with me would gladly help me, even though they have to sacrifice themselves,” “Meeting with others in games reminds me that everyone is connected in the world”).: T1:M (SD) = 3.524 (0.559), Cronbach’s α = 0.914; T2: M (SD) = 3.633 (0.604), Cronbach’s α = 0.925; T3: M (SD) = 3.655 (0.612), Cronbach’s α = 0.934.

*Loneliness* was measured using Russell’s (1996) UCLA loneliness scale (Russell, 1996). Using a questionnaire of 10 items on loneliness with answers measured on a 4-point scale (1 = not at all; 4 = all the time), 8 items were selected through a correlation and reliability analysis (e.g., “I feel a lack of camaraderie with my friends”):. T1: M (SD) = 1.620 (0.559), Cronbach’s α (AVE) = 0.929 (0.629); T2: M (SD) = 1.651 (0.604), Cronbach’s α (AVE) = 0.936 (0.660); T3: M (SD) = 1.673 (0.604), Cronbach’s α (AVE) = 0.929(0.628).

*Depression* was measured using the Center for Epidemiological Studies-Depression scale (CESD scale) developed by the National Institute of Mental Health (NIMH). Using a questionnaire of 11 items (CESD-11) on depression with respondents measured on a 4-point scale (0 = extremely rare; 3 = most of the time), 7 items were selected through a correlation and reliability analysis (e.g., “I’m very depressed”).: T1: M (SD) = 0.354 (0.457), Cronbach’s α (AVE) = 0.872 (0.509); T2: M (SD) = 0.347 (0.513), Cronbach’s α (AVE) = 0.893 (0.558); T3: M (SD) = 0.357 (0.490), Cronbach’s α (AVE) = 0.889 (0.545).

*Life satisfaction* was measured using Satisfaction with Life Scale (SWLS; Diener et al., 1985). Respondents were presented with a total of 5 questions (e.g., “My life now is close to the life I hoped for”), and answers were measured on a 5-point scale (1 = not at all; 5 = very much so).: T1: M (SD) = 4.175 (1.213), Cronbach’s α (AVE) = 0.910 (0.678); T2: M (SD) = 4.091 (1.247), Cronbach’s α (AVE) = 0.910 (0.678); T3: M (SD) = 4.183 (1.267), Cronbach’s α (AVE) = 0.916 (0.692).

*Self-esteem* was measured using the Rosenberg Self Esteem Scale (RSES; M. Rosenberg, 1965). Using a questionnaire of 10 items on self-esteem with answers measured using a 5-point scale (1 = not at all; 5 = very much so), 5 items were selected through a correlation and reliability analysis (e.g., “I feel that I have value, or at the least am equal to others): T1: M (SD) = 2.094 (0.545), Cronbach’s α (AVE) = 0.874 (0.587); T2: M (SD) = 2.090 (0.568), Cronbach’s α (AVE) = 0.873 (0.583); T3: M (SD) = 2.069 (0.583), Cronbach’s α (AVE) = 0.876 (0.585).

*Gaming time* was measured as “average daily online gaming time” among players through open-ended questions [T1, M (SD) = 38.49 min. (53.938); T2, 46.01 min. (60.837); T3, 54.43 min. (70.857)].

## Results

First, the validity and reliability of the basic statistics and measured items of the variables used in this study were investigated. The measurement invariance of life satisfaction, depression, loneliness and self-esteem was tested through factor analysis, and the eigenvalue all satisfied the fundamental assumption. In the factor analysis, the validity is secured when the eigenvalue was over 1.0. All the variables showed high validity with one component (Table 1).

**Table 1:** Number of components and Eigen value of independent variables.

Variables	Number of factors	Eigen value		
		T1	T2	T3
Life satisfaction	1	3.703	3.704	3.763
	2	0.442	0.415	0.401
Depression	1	4.040	4.327	4.272
	2	0.780	0.717	0.707
Loneliness	1	5.398	5.612	5.395
	2	0.515	0.491	0.583
Self-esteem	1	3.324	3.327	3.347
	2	0.564	0.761	0.614

T1 = the first wave, T2 = the second wave, T3 = the third wave.

Correlations and discriminant validity (Table 2) for variables by year were measured and determined to be suitable before moving on to the next steps of the study. As the AVE (average variance extracted) coefficients were above 0.5, each item’s convergent validity was verified.

**Table 2:** Correlations and discriminant validity analysis.

	G_T1	G_T2	G_T3	Satis1	Satis2	Satis3	Dep1	Dep2	Dep3	Lon1	Lon2	Lon3	SE1	SE2	SE3	SC1	SC2	SC3	SC31
G_T1	•																		
G_T2	0.622^**^	•																	
G_T3	0.576^**^	0.617^**^	•																
Satis1	−0.069	−0.021	−0.012	(0.823)															
Satis2	−0.034	−0.009	−0.081	0.555^**^	(0.823)														
Satis3	0.068	0.128^**^	0.027	0.443^**^	0.579^**^	(0.832)													
Dep1	0.087	0.006	0.017	−0.314^**^	−0.274^**^	−0.210^**^	(0.794)												
Dep2	0.128^*^	0.113^*^	0.151^**^	−0.232^**^	−0.262^**^	−0.192^**^	0.451^**^	(0.812)											
Dep3	0.164^**^	0.037	0.121^*^	−0.129^**^	−0.156^**^	−0.244^**^	0.296^**^	0.470^**^	(0.793)										
Lon1	0.022	0.018	−0.008	−0.326^**^	−0.241^**^	−0.230^**^	0.537^**^	0.261^**^	0.209^**^	(0.713)									
Lon2	0.061	0.081	0.150^**^	−0.260^**^	−0.330^**^	−0.238^**^	0.338^**^	0.610^**^	0.376^**^	0.456^**^	(0.747)								
Lon3	0.090	0.012	0.138^**^	−0.138^**^	−0.197^**^	−0.337^**^	0.249^**^	0.361^**^	0.580^**^	0.382^**^	0.545^**^	(0.738)							
SE1	−0.130^**^	−0.029	−0.083	0.343^**^	0.303^**^	0.319^**^	−0.198^**^	−0.181^**^	−0.190^**^	−0.231^**^	−0.249^**^	−0.251^**^	(0.761)						
SE2	−0.110^*^	−0.028	−0.108^*^	0.335^**^	0.466^**^	0.372^**^	−0.254^**^	−0.351^**^	−0.277^**^	−0.242^**^	−0.373^**^	−0.382^**^	0.596^**^	(0.764)					
SE3	−0.078	0.007	−0.094	0.270^**^	0.303^**^	0.511^**^	−0.241^**^	−0.327^**^	−0.362^**^	−0.257^**^	−0.332^**^	−0.404^**^	0.466^**^	0.583^**^	(0.765)				
SC1	−0.145^**^	0.002	−0.032	0.377^**^	0.200^**^	0.182^**^	−0.260^**^	−0.098	−0.108^*^	−0.457^**^	−0.261^**^	−0.176^**^	0.264^**^	0.286^**^	0.216^**^	•			
SC2	−0.065	0.038	−0.071	0.276^**^	0.340^**^	0.321^**^	−0.129^**^	−0.174^**^	−0.198^**^	−0.198^**^	−0.364^**^	−0.305^**^	0.328^**^	0.482^**^	0.340^**^	0.359^**^	•		
SC3	−0.138^**^	−0.043	−0.111^*^	0.218^**^	0.272^**^	0.322^**^	−0.160^**^	−0.166^**^	−0.233^**^	−0.220^**^	−0.295^**^	−0.361^**^	0.371^**^	0.407^**^	0.466^**^	0.337^**^	0.525^**^	•	
SC3_SC1	0.002	−0.040	−0.071	−0.130^**^	0.069	0.129^**^	0.081	−0.063	−0.113^*^	0.196^**^	−0.037	−0.168^**^	0.102^*^	0.115^*^	0.227^**^	−0.557^**^	0.156^**^	0.595^**^	•

Number in brackets: root square AVE. Off-diagonal area: correlation coefficient of each variable (^*^p < 0.05, ^**^p <. 01). G_t1 = gaming time1; G_t2 = gaming time2; G_t3 = gaming time 3; satis1 = life satisfaction1; satis2 = life satisfaction2; satis3 = life satisfaction3; dep1 = depression1; dep2 = depression2; dep3 = depression3; lon1 = loneliness1; lon2 = loneliness2; lon3 = loneliness3; SE1 = self-esteem1; SE 2 = self-esteem2; SE 3 = self-esteem3; SC1 = social capital1; SC2 = social capital2; SC3 = social capital3; t3_t1 = social capital t3-social capital.

Lastly, a repeated measures GLM analysis was conducted to examine the effect of the relationships between online game, social capital, and adolescent psychosocial factors. The results showed that there was an interaction between time and social capital (Wilks’ Lambda = 0.962, *p* < 0.01), plus there was a significant difference in adolescent game use and the transformation of psychosocial factors based on the passage of time and increase in social capital (Wilks’ Lambda = 0.868, *p* < 0.01, Table 3).

**Table 3:** Results of GLM (General Linear Model): Multivariate tests.

Effect			Value	*F*	Hypothesis df	Error df	Sig.	Partial Eta Squared
Between	Intercept	Pillai’s Trace	0.986	5712.704	5.000	397.000	0.000	0.986
		Wilks’s Lambda	0.014	5712.704	5.000	397.000	0.000	0.986
		Hotelling’s Trace	71.948	5712.704	5.000	397.000	0.000	0.986
		Roy’s Largest Root	71.948	5712.704	5.000	397.000	0.000	0.986
	SC groups	Pillai’s Trace	0.038	3.136	5.000	397.000	0.009	0.038
		Wilks’s Lambda	0.962	3.136	5.000	397.000	0.009	0.038
		Hotelling’s Trace	0.039	3.136	5.000	397.000	0.009	0.038
		Roy’s Largest Root	0.039	3.136	5.000	397.000	0.009	0.038
Within	Time	Pillai’s Trace	0.042	1.716	10.000	392.000	0.075	0.042
		Wilks’s Lambda	0.958	1.716	10.000	392.000	0.075	0.042
		Hotelling’s Trace	0.044	1.716	10.000	392.000	0.075	0.042
		Roy’s Largest Root	0.044	1.716	10.000	392.000	0.075	0.042
	Time × SC groups	Pillai’s Trace	0.132	5.941	10.000	392.000	0.000	0.132
		Wilks’s Lambda	0.868	5.941	10.000	392.000	0.000	0.132
		Hotelling’s Trace	0.152	5.941	10.000	392.000	0.000	0.132
		Roy’s Largest Root	0.152	5.941	10.000	392.000	0.000	0.132

SC groups (Social Capital groups: higher-and lower-social-capital group); Time (T1, T2, T3).

**Figure 1:**
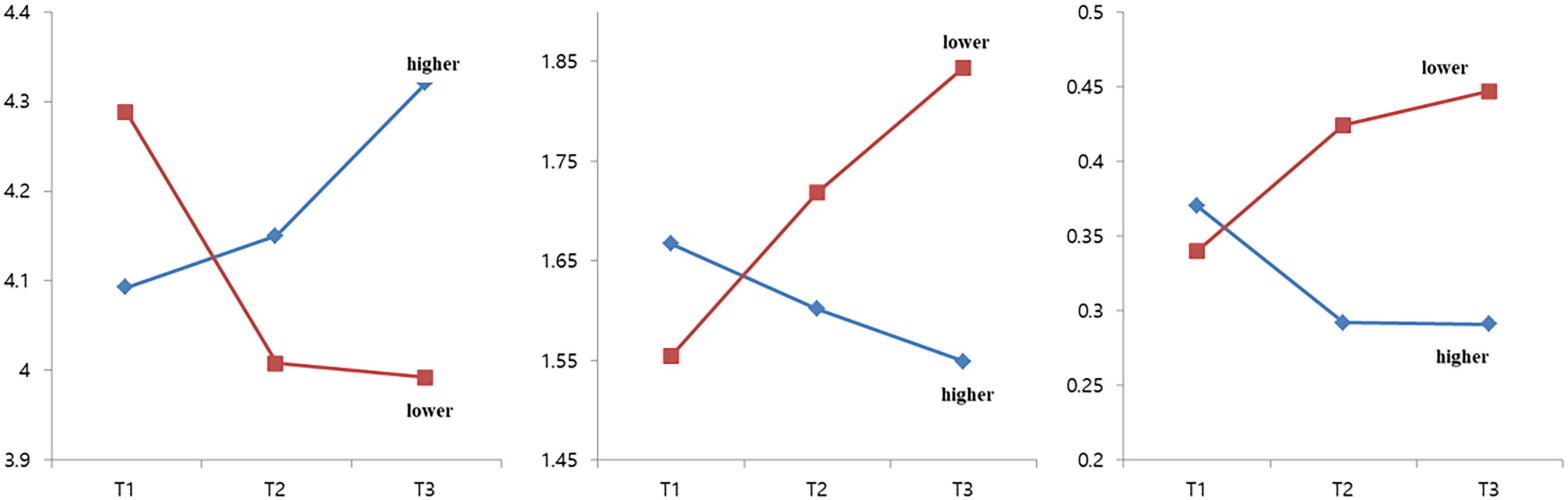
Change of life satisfaction (left), depression (middle), loneliness (right).

Based on the above results and under longitudinal circumstances, one can determine that social capital regulates the reciprocal relationship between online gameplay and time indicators of psychosocial well-being. Additionally, a more detailed analysis of the repeated measurements shows that adolescent’s life satisfaction (*F* = 9.621, *p* = 0.000), depression (*F* = 7.142, *p* = 0.001), loneliness (*F* = 23.1261, p = 0.000) and self-esteem (*F* = 3.565, *p* = 0.029) are significantly regulated by social capital (Figure 1).

Next, SEM was used to structurally examine the correlations between online game use, social capital, and adolescent psychosocial factors. With a *χ*^2^/df = 2.460, *p* > 0.05, NFI = 0.948, CFI = 0.967, TLI = 0.900, RMSEA = 0.060, the tested two-group model showed a good fit.

**Figure 2:**
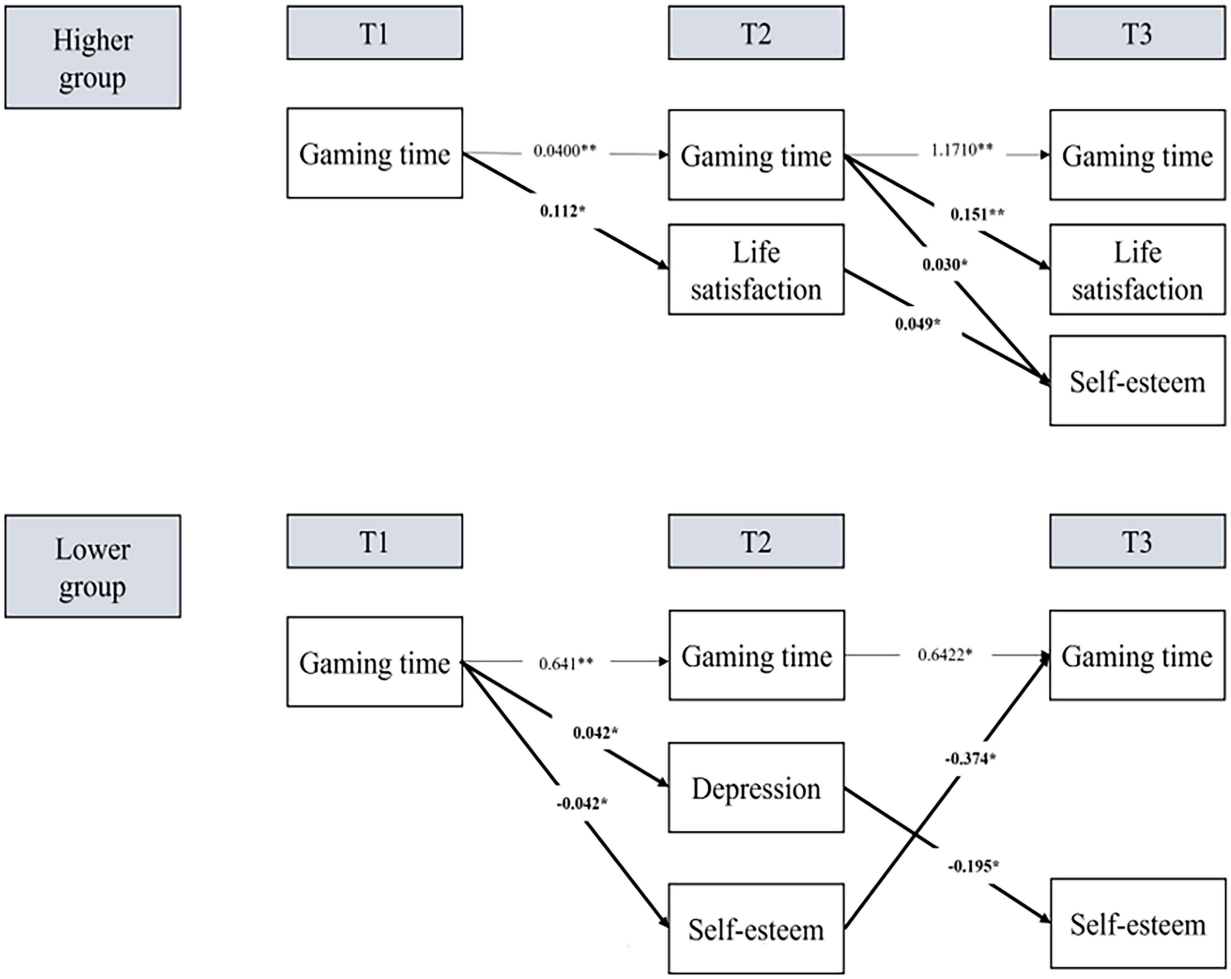
Two-group structural equation model.

Based on the above results, and under the notion that the research model presented in this study is appropriate, the cause-and-effect relationship of the reciprocal relationship between adolescent game use and psychosocial factors regarding differences based on social capital levels and the passage of time was verified (Table 4; Figure 2). In the higher social capital group, the increase of gaming time (T1 and T2) enhanced the degree of life satisfaction (T2 and T3); Life satisfaction (T2) also heightened the degree of self-esteem (T3). In the lower social capital group, gaming time (T1) increased depression (T2) and decreased self-esteem (T2); Self-esteem (T2) decreased gaming time (T3) while depression (T2) decreased the degree of self-esteem (T3).

**Table 4:** Results of hypotheses testing: Social capital lower group vs. higher group.

	T1	Lower Group		Higher Group	
		T2	T3	T2	T3
		B (C.R)	B (C.R)	B (C.R)	B (C.R)
Gaming time	gaming time	0.641^**^(8.806)	0.624^**^(12.017)	0.400^**^(5.611)	1.171^**^(5.466)
	life satisfaction	−0.020(−0.512)	0.043(1.121)	0.112^*^(2.486)	0.151^**^(4.327)
	depression	0.042^*^(1.844)	−0.014(−0.712)	−0.001(−0.057)	−0.002(−0.138)
	loneliness	0.031(1.311)	−0.003(−0.143)	0.000(0.012)	−0.026(−1.597)
	self-esteem	−0.042^*^(−2.237)	0.001(0.031)	−0.005(−0.211)	0.030^*^(1.736)
Life satisfaction	gaming time	0.011(0.093)	−0.019(−0.179)	−0.004(−0.039)	0.081(0.385)
	life satisfaction	0.396^**^(6.434)	0.533^**^(6.974)	0.595^**^(9.043)	0.530^**^(10.251)
	depression	−0.013(−0.368)	0.007(0.189)	−0.064^*^(−2.644)	0.016(0.797)
	loneliness	0.011(0.295)	0.001(0.031)	−0.082^*^(−2.624)	0.035(1.466)
	self-esteem	0.002(0.070)	0.015(0.411)	0.164^**^(4.795)	0.049^*^(1.933)
Depression	gaming time	−0.276(−0.872)	0.035(0.162)	−0.105(−0.463)	0.106(0.354)
	life satisfaction	−0.508(−2.966)^*^	−0.247(−1.554)	−0.036(−0.196)	0.203(1.076)
	depression	0.562(5.698)^**^	0.350^**^(4.418)	0.280^**^(4.150)	0.318^**^(4.404)
	loneliness	0.146(1.407)	0.007(0.088)	0.097(1.125)	−0.012(−0.137)
	self-esteem	−0.111(−1.340)	−0.195^*^(−2.653)	−0.107(−1.124)	−0.055(−0.587)
Loneliness	gaming time	−0.076(−0.272)	−0.172(−0.825)	−0.155(−0.808)	0.288(1.143)
	life satisfaction	−0.291^*^(−1.929)	−0.192(−1.295)	0.058(0.396)	0.120(0.798)
	depression	−0.037(−0.423)	0.155^*^(2.104)	0.088(1.622)	0.042(0.734)
	loneliness	0.453^**^(4.955)	0.537^**^(6.944)	0.359^**^(5.157)	0.407^**^(5.803)
	self-esteem	−0.037(−0.505)	−0.040(−0.586)	−0.320^**^(−4.194)	−0.107(−1.438)
Self-esteem	gaming time	−0.081(−0.306)	−0.374^*^(−1.817)	0.106(0.630)	−0.089(−0.429)
	life satisfaction	0.187(1.299)	−0.014(−0.093)	0.226(1.695)	0.489^**^(5.082)
	depression	−0.135(−1.631)	−0.136(−1.803)	0.035(0.712)	−0.099^*^(−2.682)
	loneliness	−0.192^*^(−2.197)	−0.220^*^(−2.789)	−0.047(−0.744)	−0.200^**^(−4.458)
	self-esteem	0.727^**^(10.463)	0.421^**^(6.019)	0.341^**^(4.884)	0.363^**^(7.595)

* *p *< 0.05;

** *p* < 0.01.

The longitudinal mediating effect was verified based on each variable and according to social capital groups. Significant indirect effects were observed from both groups, but the differences between the groups are quite profound. For the lower social capital group, indirect paths from gaming time of T1 to psychosocial factors of T3 as mediated by psychosocial factors of T2 were statistically significant: (1) gaming time → depression → self-esteem [Sobel’ Z = −2.624, *p* < 0.01; 95% bootstrap confidence interval = (−0.0903, −0.0081)], (2) gaming time → self-esteem → gaming time [Sobel’ Z = 2.055, *p* < 0.05; 95% bootstrap CI = (0.0023, 0.0395)]. For the higher social capital group, one indirect path from gaming time of T1 to self-esteem of T3 as mediated by life satisfaction of T2 (gaming time → life satisfaction → self-esteem) was statistically significant [Sobel’ Z = 2.184, *p* < 0.05; 95% bootstrap CI = (0.0059, 0.0935)].

## Discussion

### Findings

The purpose of this study was to identify the role of social capital in the effects of early adolescents’ gaming time on their psychosocial factors using a 3-wave (two years) longitudinal setting. To this end, we divided the subjects into two groups in terms of the degree of social capital (higher vs. lower), and compared the reciprocal relations among the variables in the two groups with each other. The results point to several interesting findings.

The first finding was that social capital plays a crucial mediation role. In the higher social capital group, gaming time increased the degree of life satisfaction in the first year (T1–T2), and finally enhanced both life satisfaction and self-esteem in the second year (T2–T3). However, in the lower social capital group, gaming time increased depression and decreased the degree of self-esteem in the first year. This, in turn, resulted in the decrease of self-esteem and increase in gaming time in the second year. In multivariate GLM tests, there was a significant difference in the degree of social capital between the two groups and a substantive interaction effect between time and social capital on psychosocial factors (i.e., life satisfaction, depression, and loneliness) within each group.

Second, a vicious circle was found only in the lower social capital group. In the mediation test, self-esteem (T2) strongly mediated between game play (T1) and gaming time (T3): game play ultimately increased gaming time by aggravating the degree of self-esteem. Although gaming time tend to increase in both groups, the negative loop effect of gaming time was only in the lower social capital group. The contrasting effects of gaming time in the two groups showed the importance of social capital cultivation in online games among adolescent gamers.

Third, self-esteem was the key variable differentiating the positive effect of gaming time from the negative effect among adolescent gamers. Gaming time could both increase and decrease the degree of self-esteem. In the higher group, the degree of self-esteem was enhanced with the increase in gaming time (T1–T2 and T2–T3) and life satisfaction (T2–T3). In contrast, self-esteem decreased as gaming time increased (T1–T2) and as depression increased (T2–T3) in the lower group. Considering that self-esteem is one of the critical factors to be cultivated during adolescence and online gaming has become a major part of the daily culture of adolescents, these results imply the importance of positive cultivation of self-esteem from online gaming of adolescents.

### Theoretical and practical implications

The result of this study also provided several implications. Firstly, it was the cumulative degree of social capital that determined the (positive or negative) effects of online gaming on psychosocial factors rather than gaming time. Previous studies have focused on identifying the effects of the quantitative factors of online gaming time rather than the nature of online gaming activities. Adolescents’ social capital cultivation in online games is as important as social capital formation in real life. Due to the nature of the online space, it is considered that game players, through online gaming activities, may have the opportunities to form a broader and deeper social relationship than actual reality space (Kaye et al., 2017; Gong et al., 2021). However, failure or loss of social capital development could bring about excessive gaming. Players’ emotional factors such as loneliness and depression influenced gaming time, and the degree of self-esteem mediated by social capital significantly affected the growth of gaming time. Analysis of the social support and exclusion experiences within adolescents’ online games should be prioritized.

Secondly, regarding the effects of adolescents’ gaming time on psychosocial factors, more attention should be paid to self-esteem. Since adolescent online game players who possessed poor social capital were vulnerable to exclusion from peer groups, their self-esteem, characterized by negative self-assessment and self-criticism, weakened the overall development of adolescents and made them turn to online games (Jung, 2022). Teenagers actively engaged in game activities so as not to be excluded by their peers in online games, and these endeavors led to the possibility of an increase in their online gaming time. In order to prevent gamers with poor social capital from wasting their time on games, self-esteem needs to be boosted or recovered. Several studies have confirmed that self-esteem was a moderator between social exclusion and adolescents’ life satisfaction. Reducing the adverse effects of social exclusion through positive self-assessment, lower self-criticism, and strong self-image was considerably helpful in preventing disruptive behaviors (Arslan, 2019; Banstola et al., 2020). Adequate self-esteem based on the feeling of life satisfaction among higher social capital forming players did not influence the gaming time. Thus, it is natural to infer that improving self-esteem could assist the process of social capital development.

Thirdly, the overall results of this study imply that online gaming space is as important as the real space in the cultivation of social capital for adolescents. Previous research on social capital in online games is rare, but this study focused on adolescents’ social capital in gaming experiences based on longitudinal empirical data analysis. In particular, this study emphasized that online gaming experience reflected adolescents’ developmental characteristics and social capital accumulated from the online games played an important role as peer groups of adolescence. Understanding the developmental characteristics of adolescence is necessary for research on adolescents’ online game players in that such online gameplay experience is based on virtual world play. Thus, it should not be overlooked that gaming activities could reflect real human life: cultivation of social capital in online games could be as important as social capital formation in the real world.

This study found that gaming time was not a crucial variable in terms of its effects on psychosocial factors. The lower degree of social capital and aggravated self-esteem were key variables affecting gaming time growth patterns. Peer relationships and self-esteem developed during adolescence could last for life. Therefore, online games need to be considered within the boundary of adolescents’ peer relationships and developmental self-esteem characteristics. In addition, most adolescents believe that online gaming is a combination of easy access to play with many other people and the opportunity to watch other players glean tips on how to improve their performance in a game (Clark et al., 2020). So, researchers might admit that online gameplay is the main culture of adolescents and adolescent gamers naturally long for peer support. Without fulfilling this desire, game playing tended to be long until they were satisfied with peer relationships.

This study also suggests some practical implications. Focused on the crucial role of social capital among adolescents’ gamers, game developers who want to contribute to healthy game use by adolescents could open new communities and customize events for compliments and encouragement. In addition, training programs for making good peer relationships could be helpful for excessive adolescent gamers in schools and homes. By combining online and offline social capital formation training, adolescents could have more confidence and improve game playing satisfaction levels.

In relation to the importance of self-esteem, the development of self-esteem-focused prevention and intervention programs is recommended to game policymakers. Facilitating intervention programs using in-game activities is one of the effective solutions for reducing negative effects of gaming time. Parental and youth counseling programs, physical activities programs, and arts and music lessons within local schools, communities, and counseling centers could improve the degree of self-esteem.

Finally, this study poses some limitations. The survey was conducted only in one country, South Korea, so the data used for the analysis may not reflect global characteristics. Second, more diverse variables for measuring game use behaviors could have been included. Except for gaming time, the number of games played concurrently, duration time of single play, and access time could be used as variables in assessing game use behavior. Further studies may consider these factors in longitudinal settings from diverse cultural backgrounds.

## Data availability statement

The data analyzed in this study is subject to the following licenses/restrictions: The data used in this study are available with permission from Korea Creative Content Agency (KOCCA, http://www.kocca.kr). Requests to access these datasets should be directed to EJ (stevejeong@gmail.com).

## Ethics statement

The studies involving human participants were reviewed and approved by Konkuk University IRB. Written informed consent to participate in this study was provided by the participants’ legal guardian/next of kin.

## Author contributions

GK conducted original draft preparation and data processing. EJ supervised, performed results and discussion section, and reviewed the manuscripts. JL performed literature review and discussion section. JY performed literature review and data analysis. All authors contributed to the article and approved the submitted version.

## Funding

This research was supported by Konkuk University in 2021.

## Conflict of interest

The authors declare that the research was conducted in the absence of any commercial or financial relationships that could be construed as a potential conflict of interest.

## Publisher’s note

All claims expressed in this article are solely those of the authors and do not necessarily represent those of their affiliated organizations, or those of the publisher, the editors and the reviewers. Any product that may be evaluated in this article, or claim that may be made by its manufacturer, is not guaranteed or endorsed by the publisher.

## Footnotes

^1^ www.hrc.com
